# Is movement variability altered in people with chronic non-specific low back pain? A systematic review

**DOI:** 10.1371/journal.pone.0287029

**Published:** 2023-06-14

**Authors:** Amal M. Alsubaie, Masood Mazaheri, Eduardo Martinez-Valdes, Deborah Falla

**Affiliations:** 1 Centre of Precision Rehabilitation for Spinal Pain (CPR Spine), School of Sport, Exercise and Rehabilitation Sciences, University of Birmingham, Birmingham, United Kingdom; 2 Department of Physical Therapy, Faculty of Medical Rehabilitation Sciences, King Abdulaziz University, Jeddah, Saudi Arabia; 3 Department of Plastic and Reconstructive Surgery, Erasmus MC Cancer Institute, University Medical Center Rotterdam, Rotterdam, The Netherlands; Iran University of Medical Sciences, ISLAMIC REPUBLIC OF IRAN

## Abstract

**Background:**

Variability in spine kinematics is a common motor adaptation to pain, which has been measured in various ways. However, it remains unclear whether low back pain (LBP) is typically characterised by increased, decreased or unchanged kinematic variability. Therefore, the aim of this review was to synthesise the evidence on whether the amount and structure of spine kinematic variability is altered in people with chronic non-specific LBP (CNSLBP).

**Methods:**

Electronic databases, grey literature, and key journals were searched from inception up to August 2022, following a published and registered protocol. Eligible studies must investigated kinematic variability in CNSLBP people (adults ≥18 years) while preforming repetitive functional tasks. Two reviewers conducted screening, data extraction, and quality assessment independently. Data synthesis was conducted per task type and individual results were presented quantitatively to provide a narrative synthesis. The overall strength of evidence was rated using the Grading of Recommendations, Assessment, Development and Evaluation guidelines.

**Findings:**

Fourteen observational studies were included in this review. To facilitate the interpretation of the results, the included studies were grouped into four categories according to the task preformed (i.e., repeated flexion and extension, lifting, gait, and sit to stand to sit task). The overall quality of evidence was rated as a very low, primarily due to the inclusion criteria that limited the review to observational studies. In addition, the use of heterogeneous metrics for analysis and varying effect sizes contributed to the downgrade of evidence to a very low level.

**Interpretation:**

Individuals with chronic non-specific LBP exhibited altered motor adaptability, as evidenced by differences in kinematic movement variability during the performance of various repetitive functional tasks. However, the direction of the changes in movement variability was not consistent across studies.

## Introduction

Low back pain (LBP) affects the majority of the worldwide population at some point during their lifetime [1, 2]. Understandably, LBP can have a substantial effect on the quality of life of those affected [3, 4].

People with LBP move differently than pain free individuals [5, 6]. However, the underlying mechanisms of motor adaptations to pain still require further clarification at both the micro (e.g. single motor neuron) and macro (e.g. whole muscle behaviour) levels [6]. One common motor adaptation to pain is changes in spine kinematics, such as angular displacement, angular velocity, smoothness, and variability of movement [6–8]. Variability of movement is a common feature of human movement, especially during routine daily activities requiring repetitive (cyclic) movements [9, 10]. As an example, one study showed that people with LBP show more variability in coordination between the spine and pelvis during sit-to-stand, which is likely a strategy to find an optimal pain-free pattern [11].

Several theories have been proposed to describe natural variability in motor performance [12–14]. Some theories propose that decreased variability generally indicates a highly stable and coordinated behaviour [10]. Increased variability could also be considered as a beneficial component for motor performance, as it may reduce the risk of injury by distributing joint loads [15]. However, it is not well established how trunk movements should ideally be performed in terms of the adequate amount of motor variability, stability and complexity to achieve a coordinated movement that doesn’t predispose an individual to injury, specifically during dynamic and repetitive tasks [16].

Changes in motor variability in people with LBP has been assessed in various ways, including assessing the variability of kinetic and kinematic measures, ‘coordinative’ aspects of a movement pattern, as well as muscle activity patterns [17]. However, it remains unclear whether movement in people with LBP is typically characterised by increased, decreased, or unchanged variability [18–21]. One possible reason for this is the large variation seen across individuals with LBP [22]. Another possible reason is the use of various linear and non-linear measurement tools to quantify the amount and structure of motor variability, respectively [23–25]. Both the amount and structure of variability functionally represent different aspects of motor performance and can vary independently from one another [23].

Systematic reviews have examined biomechanical and neuromuscular changes in people with LBP, and to a lesser extent, the motor variability during specific functional tasks, such as gait and quiet standing [26, 27], or balance performance for a specific age category, i.e. elderly [28]. However, only scoping reviews have examined motor variability in the presence of spinal pain [20, 21]. Both reviews revealed large heterogeneity between study designs and results. Dijk et al. 2021, attributed the inconsistent findings between studies to differences in normalization methods, changes in functional tasks, and differences in pain intensity or fear-avoidance behaviours of those with LBP [20]. Although Dijk et al. 2021 included participants with chronic LBP, motor variability was assessed based on electromyography (EMG) recorded from the thoracolumbar region and kinematic movement patterns in all regions (e.g., including the knee) during functional tasks.

To date, no systematic review has synthesised findings on kinematic variability of the spine in people with chronic non-specific LBP during the performance of repetitive functional tasks. Therefore, the aim of this systematic review was to synthesise the evidence on whether the amount and structure of motor variability in kinematics (such as joints displacement, acceleration, and velocity) are altered in people with chronic non-specific LBP at the thoraco-lumbar and/or lumbo-pelvic regions.

## Methods

This systematic review was planned according to the updated guidelines of the Cochrane Back and Neck Group [29], Cochrane Handbook for Systematic Reviews of Interventions [30], and the review is reported following the Preferred Reporting Items for Systematic Reviews and Meta-Analyses (PRISMA) statement **(S1 Table)** [31]. The protocol was registered a priori with PROSPERO, Centre for Reviews and Dissemination, University of York, (CRD42020211580) on 10/12/2020, and the protocol was published in advance [32].

### Eligibility criteria

Eligibility criteria were defined with the PICOS framework (P: Population; I: Indicator/ Exposure; C: Comparator; O: Outcome(s); S: Study design) [30, 33]. Eligibility criteria are summarized in (**Table 1)**.

**Table 1:** Summary of inclusion and exclusion criteria.

***Inclusion criteria***	
**Population**	Adults (≥18 years old), men and women with chronic non-specific LBP
**Indicator/Exposure**	Motion analysis systems (e.g. optoelectronic systems, inertial measurement unit sensors, etc.)
**Comparison**	Healthy controls
**Outcomes**	Amount or structure of kinematic variability based on linear or non-linear measures
**Study Type**	Quantitative cross-sectional observational studies
***Exclusion criteria***	
**Population**	Individuals under the age of 18, people with LBP attributable to a specific pathology, concurrent systemic disorders, surgery, cardiovascular conditions or pregnancy
**Indicator/Exposure**	None
**Comparison**	Individuals under the age of 18, concurrent systemic disorders, surgery, cardiovascular conditions or pregnancy
**Outcomes**	Studies using spatio-temporal parameters based on neuromuscular variables (i.e. EMG signals) only
**Study Type**	Cadaveric or animal studies, single-subject case reports, longitudinal cohort studies, interventional studies including both randomized and non-randomized clinical trials, reviews, meta-analysis and study protocols as well as studies not written in English.

### Population

The population of interest were men and women with an age of 18 years and above with chronic non-specific LBP that persisted for at least 3 months with no diagnosable underlying pathology [34]. They did not present with concurrent systemic disorders including rheumatic and neuromuscular disorders, spinal deformity or surgery, cardiovascular conditions and pregnancy. Studies that recruited people with LBP due to trauma, fractures, spinal stenosis, or radicular pain were excluded from this review.

### Indicator

Studies were considered eligible if they used motion capture systems (e.g. optoelectronic systems, inertial measurement unit sensors, etc.) to quantify variability of spinal movement at the level of thoraco-lumbar and/or lumbo-pelvic regions [23, 35].

#### Comparison

For the purpose of this review, adults without a history of LBP represented the control group. No concurrent systemic disorders including rheumatic and neuromuscular disorders, spinal deformity or surgery, cardiovascular conditions and pregnancy were present in the participants.

#### Outcomes

The main outcome of interest was kinematic motor variability, either the amount or structure measured based on a wide range of movement variables during repetitive functional tasks. Multiple kinematic outcome measures that quantified spinal movement at thoraco-lumbar and/or lumbo-pelvic regions were considered for the variability measures (i.e., amount or structure) including joint displacement (or range of motion), acceleration, velocity, coordination (a measure between two kinematic variables).

#### Study design

Observational cross-sectional studies were included. The following study types were excluded: cadaveric or animal studies, single-case studies, longitudinal cohort studies, and interventional studies including both randomized and non-randomized clinical trials, reviews, meta-analysis and study protocols as well as studies not written in English.

### Information sources and search strategy

The following databases were searched initially from inception to 14^th^ December 2020 by one reviewer (AMA); updated up to 23^rd^ August 2022 by the same reviewer (AMA): MEDLINE (OVID Interface), EMBASE (OVID Interface), CINAHL (EBSCO Interface), ZETOC (EBSCO Interface), Web of Science, PubMed and Scopus. Reference lists of included studies and relevant reviews were checked. Moreover, hand searching was conducted of relevant key journals including: Journal of Electromyography and Kinesiology, Clinical Biomechanics, Journal of Biomechanics, Human Movement Science, The Clinical Journal of Pain, Spine, Journal of Orthopaedic & Sports Physical Therapy, Musculoskeletal Science and Practice and Journal of Back & Musculoskeletal Rehabilitation.

The search strategy was developed based on the PICOS framework using medical subject headings (MESH) identified during the scoping search (**Table 1)**. A complete search strategy example used in MEDLINE (OVID interface) was provided in the published protocol [32] **(S1 File)**. Appropriate modifications with relevant syntax and MESH terms was performed to the main search strategy to adapt for other databases and ensure consistency. Grey literature was included in the search using the British National Bibliography for Report Literature (BNBRL), OpenGrey database, ProQuest Dissertations & Theses Global and EThOs to reduce the risk of publication bias. Key congresses and meetings in the field were searched from 2017 to 2022, including the World Congress of Biomechanics (WCB) and the International Society of Electrophysiology and Kinesiology (ISEK) congresses.

### Study selection

Records were retrieved from databases and imported into EndNote V.X9 (Clarivate Analytics). After the removal of duplicates, eligible studies were selected by two reviewers (AMA, MM) who independently conducted title and abstract screening of all retrieved studies against the predetermined eligibility criteria and categorised articles into ‘include’, ‘unsure’ (e.g., needs full text review) and ‘exclude’. Eligible full-text studies were screened by one reviewer (AMA) and the selection decision was confirmed by the second reviewer (MM). Any disagreement between the reviewers in the study selection process was resolved by discussion.

### Data extraction and data items

One reviewer (AMA) extracted the data from the eligible studies using a customised data extraction form and then the accuracy was confirmed by a second reviewer (MM). A third reviewer (DF) was available to mediate any disagreements. WebPlotDigitizer software was used to extract data from figures when text and tables were not sufficient to obtain the main outcome results. The following components were extracted: Population (inclusion/exclusion criteria, sample size and demographic, i.e., sex, age, height, weight, body mass index, and clinical characteristics, such as pain intensity and duration, disability level, fear, and other psychological factors; Indicator (type of task performed, measurement instrument, and spine region assessed); Outcome (variability measure/metrics); and Results (mean and SD of outcome measures and their statistical significance.

### Risk of bias assessment

Due to the lack of a gold-standard instrument designed to quantify the quality of observational cross-sectional studies, the methodological quality of the included studies was assessed using a modified version of Downs and Black Scale (D&B), designed for assessing the quality of both randomized and non-randomized studies [36]. Among various tools available, D&B is the most commonly used tool to assess the methodological quality of observational studies [37]. This was done independently by two reviewers (AMA, MM). The modified version consists of 4 domains (12 items) including quality of reporting (7 items), the generalizability of results or external validity (1 item), the relationship between LBP and outcomes, known as internal validity (3 items) and the adequacy of sample size or study power (1 item). Items 4, 5, 6 and 11 were specifically implemented from a previous review to fit the study design of the current review [38]. The scoring was simplified to a choice of 0 (“no”/“unable to determine”) or 1 point (“yes”). Therefore, the total scores range from 0 to 12 and higher scores indicate a better methodological quality of the study [39, 40]. However, for the purposes of this review, the total score was presented with a percentage score; moderate quality when 60–74% of the applicable criteria was met; >75% as high and <60% as low quality [41, 42].

### Summary measures and data synthesis

Due to the heterogeneity of the studies (i.e., nature of the task, spine region, the used variability measure and metrics), meta-analysis was not possible and therefore a narrative synthesis was conducted. The studies were sub-grouped according to the nature of the task preformed during the assessment. In contrast to previous reviews [20, 21], visualization of quantitative data was considered important in this study and hence effect estimates and their confidence interval for each study was presented in a forest plot without producing the overall estimate of effect. Results were reported using mean and standard deviation (SD). When standard error (SE) was reported, SD was calculated using the formula: SD = SE*√N; N: sample size [43]. When the LBP population was presented as subgroups (i.e. male/female, low disability/high disability, athletes/non-athletes, or different pain intensity levels 1–10), we followed the Cochrane guidelines handbook and used approporiate formulae for combining groups [43]. Differences between the LBP and control groups were summarised using the standardised mean difference (SMD) and 95% confidence intervals (95% CI). Forest plots were produced in RevMan software (v.5.4 Cochrane Collaboration) [44].

### Quality of evidence

The quality of evidence was assessed for the main outcome domain per task type using the Grading of Recommendations Assessment, Development, and Evaluation (GRADE) approach [45]. The level of evidence was then graded by each reviewer independently where ratings were made as per `high’, `moderate’, `low’ or `very low’ [46]. Initially, since only observational studies were included, low quality of evidence was assigned to the primary outcome domain per task type preformed [47]. Then, the quality of evidence was rated considering five factors (limitations, inconsistency, indirectness, imprecision, publication bias) [47]. The D&B individual score for each study was integrated into the GRADE approach to define the study limitations of evidence (for each outcome domain) [48]. Therefore, if the evidence was mainly obtained from studies with low methodological quality (D&B score <60%), limitations were described as serious. No limitation was reported with moderate methodological quality studies (D&B range from 60 to 74%). Finally, the level of quality of evidence was upgraded when most of the findings came from studies with high methodological quality [48]. Overall, the level of evidence was identified as ‘High’, ‘Moderate’, ‘Low’, or ‘Very Low’ [47].

## Results

### Study selection

**Fig 1:**
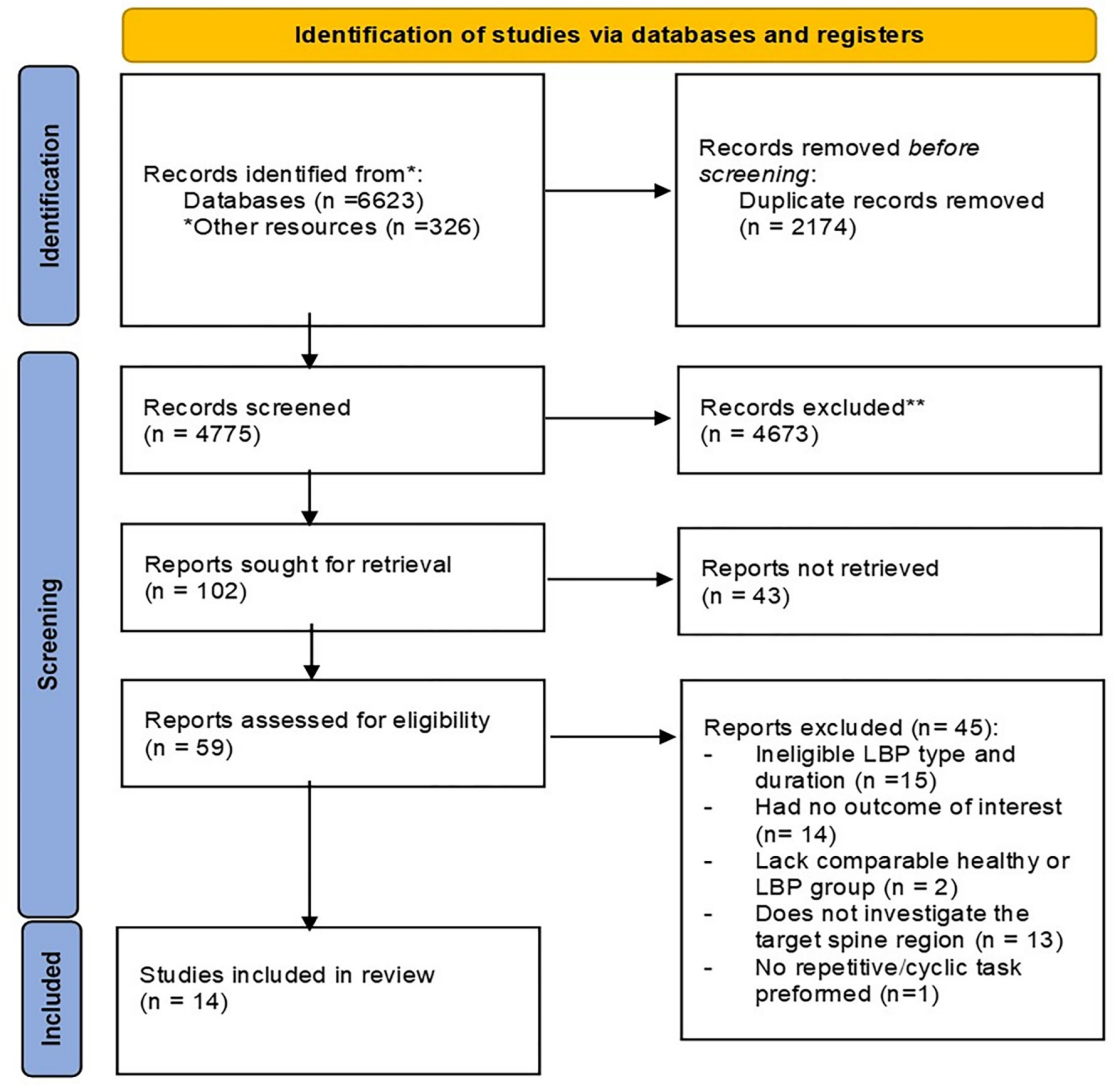
Preferred Reporting Items for Systematic reviews and Meta-Analyses (PRISMA) 2020 flow diagram. *Other resources that were identified through searching the gray literature and hand search.

The database search retrieved a total 6949 records that were identified through both database and hand searching. After the removal of duplicates, 4775 records were title and abstract screened by the two reviewers. A total of 59 studies were retained for full text screening. Finally, 14 studies were obtained for quantitative narrative synthesis. The search strategy and reasons for exclusion (**S2 File)** are provided in the PRISMA flow chart in **Fig 1**.

### Characteristics of included studies

All of the 14 included studies were observational studies published between 2001 and 2022 [19, 49–61]. In total, 380 people with chronic non-specific LBP were included (women: 41%; men: 55% except one study that did not mention the women/men ratio [59]) and 276 healthy people. The mean age of LBP participants ranged from 20.8 to 44.1 years with average pain intensity of 3.1 out of 10 on a numerical pain scale and an average pain duration of 34.7 months. The average disability level was 17.5% in studies that used Oswestry-disability-index (ODI) and 5.3 in studies that used Roland–Morris Disability Questionnaire (RDQ), indicating a minimal level of disability. Only three studies [19, 55, 57] assessed the fear-avoidance behaviour and beliefs using Tampa Scale for Kinesiophobia (TSK), with an average score of 30.9 indicating moderate levels of fear and avoidance [62], and only two [19, 55] measured catastrophic thinking related to pain using the Pain Catastrophizing Scale (PCS), with an average score of 11.25.

To facilitate the interpretation of the results, the included studies were grouped into four categories based on the task preformed. Detailed descriptions of the inclusion/exclusion criteria **(S2 Table),** and demographic and clinical characteristics of the included studies are presented in **Table 2**.

**Table 2:** Demographic and clinical characteristics of included studies.

First Author	Sample size; sex (f/m); age (yrs); height (cm); weight (kg); BMI (kg/m^2^)			LBP characteristics		
	LBP	Healthy control	Matching criteria for two groups	Intensity; duration	Disability level	Psychological status
**1. Repeated Flexion and Extension task**						
Graham et al. 2014 [49]	10; 6/4; 20.8 (1.4); 175.9 (14.0); 71.8 (14.1); NR	10; 6/4; 20.6 (1.0); 173.7 (11.1); 72.5 (12.0); NR	Sex; age; height; weight	NR; 24.4±23 months	ODI (0–50): 7.8 (3.5); RMDQ (0–24): 4.0 (2.1)	NR
Mokhtarinia et al. 2016 [50]	22; 0/22; 30.2 (6.1); 177 (7.7); 74.5 (7.7); 23.9 (3.3)	22; 0/22; 27.4 (5.1); 174 (8.2); 71.4 (10); 23.5 (3.5)	Age; height; weight	VAS (0–10) < 2; NR	NR	NR
Bauer et al. 2017 [51]	59; 29/30; 39.1 (12.8); NR; NR; 24.0 (3.6)	27; 15/12; 39.6 (11.6); NR; NR; 22.7 (2.8)	NR	NRS ≥ 1; NR	ODI > 8% moderate disability	STarT Back Screening Tool- psychological subscale: < 4
**2. Lifting task**						
Dideriksen et al. 2014 [19]	17; 10/7; 32.5 (9.6); 177. 4 (9.6); 74.3 (12.8); NR	17; 9/8; 29.7 (7.3); 174.8 (10.3); 69.2 (14.0); NR	Sex; age	NRS: 3.1 (2.2); 34.2 (29.3) months	ODI: 14.2% (7.2)	SF-36 (total) (0–100): 66.9 ± 12.2 Physical (0–100): 60.9 ± 14.2 Mental (0–100): 67.6 ± 14.1 TSK (17–68): 31.8 ± 5.9 PCS (0–52): 16.1 ± 8.5 STAI (20–80): 40.2 ± 7.1
Bauer et al. 2015 [52]	63; 31/32; 39.2 (12.5); NR; NR; 24.2 (3.9)	31; 17/14; 40.1(12.1); NR; NR; 22.7(2.9)	NR	NRS: 3.3 (1.5) ;NR	ODI > 8%, moderate disability	SBT psychosocial subscale (≥4 high risk): < 4
Moreno Catalá et al. 2018 [53]	*Non-athletes*: 15; 5/10; 27 (1); 178 (7); 78 (17.7); 24.3 (4.3) *Athletes*: 15; 5/10; 23 (2); 175 (10);72.9 (10.7); 23.6 (1.6) *Combined*: 30; 10/20; 25 (2.6); 176.5 (8.6); 75.5 (14.6); 23.9 (3.2)	*Non-athletes*: 14; 5/9; 24 (3); 175 (11); 70.3 (11.2); 22.7 (2.5) *Athletes*: 15; 5/10; 23 (3); 178 (9); 73.4 (13.4); 22.8 (2.4) *Combined*: 29: 10/19; 23.5 (2.9); 176.5 (9.9); 71.9 (12.2); 22.8 (2.4)	Age; height; weight	VAS: 3.92±1.70 (Non-athletes) , 4.54±1.82 (athletes), 4.2 ±1.8 (combined); ≥ 12 months	NR	NR
Pranata et al., 2018 [54]	*High disability (ODI > 20%)*:18; 12/6; 46.7 (11.8); 170 (10); 71.5 (14.0); 24.8 (3.8) *Low disability (ODI < 20%)*: 25; 11/14; 42.3 (11.1); 174 (10); 81.6 (19.0); 26.0 (5.0) *Combined*: 43; 23/20; 44.1 (11.5); 172.3 (10); 77.3 (17.6); 25.5 (4.5)	29; 17/12; 37.8 (11.5); 167 (10); 73.4 (17.6); 25.9 (5.6)	Sex; age; BMI	NRS (0–10): 4.5 ± 1.9 (high disability, 3.0 ± 1.6 (low disability), 3.6 ± 1.9 (combined) ; 155.2 ± 173.2 months (high disability), 110.2 ± 107.8 months (low disability), 129 ± 138.8 (combined)	ODI (0–100%): 34.4 ± 10.9 (high disability)13.2 ± 4.9 (low disability); 22 ±13.2 (combined)	NR
Fujii et al., 2022 [55]	31; 0/31; 30.5 (6.0); 172.7 (3.6); 65.5 (4.2); NR	20; 0/20; 28.1 (5.2); 172.0 (4.8); 66.7 (9.0); NR	NR	NRS in the past 4 wks (0–100): 3.6 (1.7); 14.7 (14.9) months	RMDQ (0–24): 2.1 (1.4)	TSK-11 (11–44): 21.9 (5.0). PCS-4 (0–16): 6.4 (3.9). Fre-BAQ (0–36): 7.5 (6.5)
**3. Gait**						
Vogt et al. 2001 [56]	*Male*: 21; NA; 36.3 (1.7); 173.3 (9.1); 77.8 (16.3); 25.8 (4.1) *Female*: 13; NA; 32.1 (3.4); 175.9 (5.6); 76.9 (12.5); 24.8 (3.3) *Combined*: 34; NA; 34.7 (3.2); 174.2 (7.9); 77.5 (13.1); 25.4 (3.7)	*Male*: 16; NA; 34.8 (5.2); 178.6 (4.7); 77.6 (6.5); 25.5 (2.4) *Female*: 6; NA; 29.4 (1.3); 170.3 (9.9); 71.5 (4.9); 23.7 (2.6) *Combined*: 22; NA; 33.3 (5.0); 176.3 (7.3); 75.9 (6.6); 25.0 (2.5)	NR	VAS (0–10): 3.7 (range, 3–5.3); NR	ODI: 27.67%; (range, 24–48%)	NR
Lamoth et al. 2006a [57]	19;11/8; 38 (21–52); 173 (154– 188); 74.4 (49–97); NR	14; 5/9; 31 (20–46); 180 (158–198); 72.5 (52–105); NR	Sex; age; height; weight	VAS: 5.6 (3); 14.4 (3.5–36) months	RDQ: 10 (6)	TSK: 39 (6.8)
Lamoth et al. 2006b [58]	12; 7/5; 36.8 (10.9); 174 (11); 72.4 (14.5); NR	12; 5/7; 30±8.1; 180 (12); 73.3 (16.6); NR	Age; height; weight	NR; NR	NR	NR
Seay JF et al. 2011 [59]	14; NR; 35.71 (10.90); 171 (12); 73.94 (13.42); NR	14; NR; 29.90 (8.45); 169 (10); 63.94 (10.06); NR	Age; height; weight	VAS (1–10): 0.8 (1.4); NR	ODI (0–100%) : 7.9% (6.3)	NR
Ebrahimi et al. 2017 [60]	10; 5/5; 29.4 (6.38); 68.07 (12.92); 164.4 (8.19); NR	10; 5/5; 29.6 (5.64); 62.38 (13.12); 167.40 (7.36); NR	Sex, age, height, and weight	NRS: 5.1 (0.88); NR	ODI: 37.30% (12.66)	NR
**4. Sit to stand to sit (STS) task**						
Ippersiel et al. 2018 [61]	16; 11/5; 30 (9); NR; 78.6 (18.5); NR	21; 10/11; 27 (10); NR; 67.4 (10.0); NR	NR	NPRS (0–10): 3.4 (1.1); 109.9 (113.5) months	ODI: 25.3%(7.4)	NR

### Narrative synthesis of results

The main findings of each study are summarised in **Table 3** with more detailed description of the task preformed provided in **S3 Table.** The outcome measures of interest used in the eligible studies were drawn from either linear or non-linear metrics or both. The studies exhibited a significant degree of heterogeneity with respect to the spinal regions examined, the type of the tasks preformed, and the metrics used to analyse data. Therefore, meta-analysis was inappropriate and individual results are presented quantitatively using RevMan. The interpretation of the reported effect sizes were based on the mean difference where (0.0–0.2) considered small effect, (0.4–0.5) medium, and (0.8–3.0) large effect [63].

**Table 3:** Measurement protocol of included studies.

First Author	Task preformed	Instrument used	Spine region assessed	Outcome measures	*P* value
**1. Repeated Flexion and Extension task**					
Graham et al. 2014 [49]	Two randomized trials, symmetric and asymmetric, of 30 repetitive and continuous trunk flexion and extension movements from standing position, with a constrained pelvis and their hands held together	Electromagnetic motion sensors (Liberty, Polhemus, Colchester, VT, USA)	Lumbar spine (over T12 and S1)	Maximum Lyapunov exponent, λmax **(**Local dynamic spine stability)	**Dynamic stability:** **Group:** *p* = 0.440
Mokhtarinia et al. 2016 [50]	30 cycles of repeated trunk flexion–extension (touching target placed at knee level followed by returning to upright position)	3D video-motion analysis system (VICON, Oxford, UK)	Lumbar–pelvis (T10- PSIS-ASIS)	Deviation phased DP (sagittal plane)	**DP (Degree):** **Group:** *p* = 0.75
Bauer et al. 2017 [51]	Repeated trunk flexion and extension test in sitting position	Inertial measurement unit (IMU) system (Valedo ®Motion, Hocoma AG, Volketswil, Switzerland)	Lumbar between 1^st^ lumbar and 2^st^ sacral vertebra	Determinism% and Sample entropy of angular displacement and velocity	**Pre-Fatigue condition:** **Angular Displacement** **Group:** **%DET:** *p* = 0.138 **SaEn**: *p* = 1.0 **Angular velocity** **Group:** **%DET:** *p* = 0.57 **SaEn**: *p* = <0.001*
Dideriksen et al. 2014 [19]	Repetitively move a box (5 kg) between two shelves, located at knee and shoulder height	Epionics SPINE (Epionics Medical GmbH, Potsdam, Germany) with two sensor strips, each with 12 evenly spaced angle sensors (25 mm apart from one another); The bottom of the strip located at the posterior superior iliac spine (PSIS)	From PSIS 30 cm upward covering the whole spine	Determinism%	**%DET** **Group:** Average *p* = 0.54
**2. Lifting task**					
Bauer et al. 2015 [52]	Pick up a box from standing position, 10 cycles	Inertial measurement unit IMU system (ValedoMotion, Hocoma AG, Volketswil, Switzerland)	Lumbar between 1^st^ lumbar and 2^st^ sacral vertebra	Recurrence (REC) and Determinism (DET) of angular displacement, velocity, and acceleration	**Angular displacement (AD)** **Group:** REC AD: *p* = 0.018 DET AD: p = 0.01* Angular velocity (AV) REC AV: *p* = 0.03* DET AV: *p =* 0.05* Angular acceleration (AA) REC AA: *p =* 0.03* DET AA: *P =* 0.05*
Moreno Catalá et al. 2018 [53]	Lifting task, in which a pot (1.5 kg) was cyclically moved back and forth between two tables of different heights (40 cycles); participant standing in the middle of two tables	Vicon 624 system (Vicon Motion Systems, United Kingdom)	Lumbar-pelvis: Region between L4/L5 disk space and ninth thoracic vertebrae.	Maximum Lyapunov exponent (λmax)	**Dynamic stability (λmax):** **Group:** *p =* 0.136
Pranata et al., 2018 [54]	Lifting an 8-kg kettlebell up to the level of their abdomen using a self-selected pace and technique; repeated twice	12-camera Optitrack Flex 13 motion analysis system (NaturalPoint, Corvallis, OR)	Lumbar-pelvis in the sagittal plane	Deviation phase (DP)	**DP lumbar-hip (Degree):** **Group:** *p =* 0.13
Fujii et al., 2022 [55]	Lifting a box (520 × 365 × 305 mm) placed on the ground as quickly as possible to waist-height. In three different conditions	A three-dimensional (3D) motion capture system with a four-charge-coupled device (CCD) camera (KinemaTracer, KisseiComtec, Matsumoto, Japan).	Upper and lower lumbar regions. (L3 spinous process and S1 spinous process)	Deviation phase (DP)	**DP (Degree):** **Flexion phase:** **Group:** *p =* 0.10 **Extension phase:** **Group:** *p =* 0.57
**3. Gait**					
Vogt et al. 2001 [56]	Walking on a motorized treadmill at 4.5 km/h; data from a 30-second interval recorded while walking for approximately 3 minutes	Treadmill: a motorized treadmill (HPCosmos, Germany); Motion: three-dimensional ultrasonic movement analysis system (Zebris CMS 70, Germany)	Thoracolumbar (markers placed on T12 and S1)	Coefficient of variation (CV)	**Stride-to-Stride** **CV:** **Group:** *p*< 0.001*
Lamoth et al. 2006a [57]	Walking on a treadmill at a self-selected walking velocity succeeded by increasing velocity from 1.4 km/h to a maximally attainable walking velocity of up to 7.0 km/h	3D active marker movement registration system (Optotrak 3020, Northern Digital, Ontario, Canada)	T3, L2 spinous processes, and the sacrum (between posterior superior iliac spine PSIS)	SD relative phase between lumbar and pelvis rotations (transverse and frontal plane)	**Relative phase variabilit (Degree):** **Group:** Transverse plane: *p =* 0.04* Frontal plane: *p*<0.01*
Lamoth et al. 2006b [58]	Walking on a treadmill at six velocities in a fixed order: 6.2, 1.4, 3.8, 5.4, 2.2, and 4.6 km/h	3D active marker movement registration system (Optotrak 3020, Northern DigitalTM, Ontario, Canada)	Third thoracic vertebra (T3), second lumbar vertebra (L2), and the sacrum	SD relative phase between lumbar and–pelvis rotations (transverse and frontal plane)	**Relative phase variability (Degree):** **Group:** Transverse plane: *p =* 0.03* Frontal plane: *p*<0.01*
Seay JF et al. 2011 [59]	Walking (at various speeds systematically increased from 2.88km/h in increments of 1.8km/h to 13.68 km/h	Treadmill: (Frappier Acceleration, Fargo, ND, USA and Advanced Mechanical Technologies, Incorporated, Waltham, MA, USA); Motion: Three-dimensional kinematic data were collected using eight high-speed cameras (Motion Analysis Corp., Santa Rosa, CA)	Pelvis–trunk (T12 L1 -L5 S1 inter-vertebral joint space) in sagittal, frontal and transverse plane	SD relative phase	**Relative phase variability (degree):** **Group:** **Sagittal**: Walking: *p =* 0.662 Running:*p =* 0.122 **Frontal:** Walking:*p =* 0.402 Running:*p =* 0.985 **Transverse:** Walking:*p =* 0.203 Running:*p =* 0.022*
Ebrahimi et al. 2017 [60]	Walking barefoot along an 8-m walkway at a comfortable self-selected speed; at least 20 complete gait cycles for each limb captured	An eight-camera motion analysis system (Proreflex, Qualisys Track Manager® Ltd., Gothenburg, Sweden)	Upper and lower back–pelvis.	Deviation phase over stance and swing (sagittal plane)	**DP (degree):** **Group:** Stance: *p =* 0.049* Swing: *p* = 0.008*
**4. Sit to stand to sit (STS) task**					
Ippersiel et al. 2018 [61]	Sit to stand to sit (STS) task with arms crossed over chest, including standing upright from sitting and return to sitting as quickly as possible, 10 trials	An electromagnetic TrakSTAR motion capture system with model 800 sensors (Ascension Technology, Milton, VT, USA)	The lower lumbar spine (L3-S1) and upper lumbar spine (T12-L3)	Deviation phase DP (sagittal plane)	**DP (degree):** **L3S1-T12L3:** **Group:** *p* = 0.147

### Type of task

#### Repeated flexion and extension

**Fig 2:**
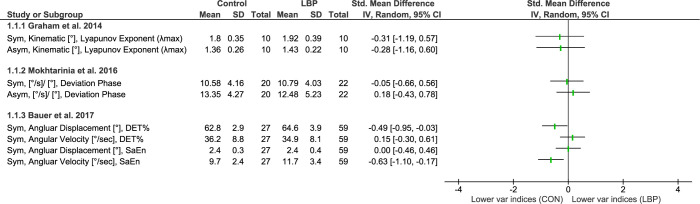
Differences in movement variability between people with LBP and healthy controls (CON) while preforming repeated flexion and extension tasks. Abbreviations: Sym: symmetric; Asym: Asymmetric; %DET: Percentage determinism; SaEn: Sample entropy; Var: Variability.

Three studies investigated movement variability during repeated flexion-extension task performed under symmetrical (n = 3) [49–51] or asymmetrical (n = 2) conditions [49, 50] (**Fig 2)**. One study used a linear measure known as deviation phase (DP) to quantify the amount of intersegmental coordination variability [50]. The other two studies employed non-linear measures, with one study characterizing the structure of variability in angular displacement using maximum Lyapunov exponent (λmax) [49], and the other study assessing the structure of variability in both angular displacement and velocity using percentage determinism (DET%), and sample entropy (SaEn) [51]. It is worth noting that λmax was defined as a measure of local stability, where lower λmax values is associated with higher stability [49], while DET% was described as a measure of predictability and SaEn as a measure of complexity [51].

Out of a total of eight measurements, only two measures from one study showed a significant difference in variability indices between people with and without LBP [51]. Those with LBP showed a higher level of complexity and less predictability in angular velocity (as indicated by high SaEn), but greater predictability in angular displacement (as indicated by high DET%). A more pronounced effect was observed for angular velocity (with a medium effect size) compared to angular displacement (with a small effect size). However, this effect was not reported in the study, as the main comparison focused on detecting the effect of fatigue using a linear mixed model. Therefore, the effect of pain was only measured based on the pre-fatiguing condition using the reported means, SDs, and sample sizes, which was significant only for SaEn on angular velocity (*p* = <0.001). Overall, only one of the three studies reported a small to medium effect of LBP status on variability measures during the repetitive trunk flexion and extension task, indicating a reduction in predictability and an increase in complexity.

#### Lifting

**Fig 3:**
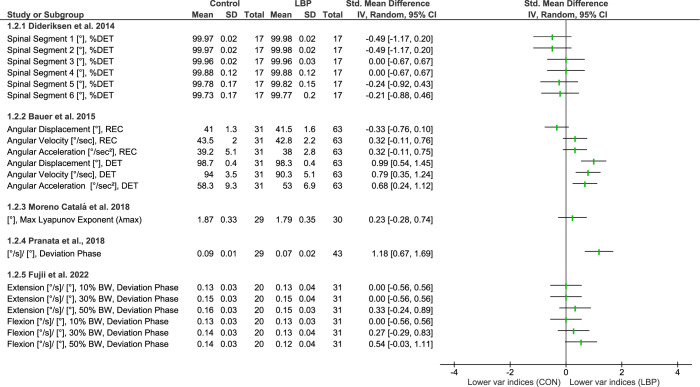
Differences in movement variability between people with LBP and healthy controls (CON) while preforming lifting tasks. Abbreviations: DET: Determinism; REC: recurrence rate; Var: Variability.

Five of the included studies investigated movement variability during lifting tasks. Two of these studies [54, 55] used a linear measure, specifically DP, while the remaining three studies [19, 52, 53] utilized non-linear measures, including recurrence rate (REC), DET, and λmax (**Fig 3)**.

All studies, apart from one [52], did not show differences for people with LBP for movement variability during lifting. Bauer et al. 2015 showed less predictable lumbar movement patterns, as indicated by a significant reduction in determinism for various movement variables, including angular displacement, velocity, and acceleration [52].

Even though the forest plot in the study of Pranata et al. 2018 showed a significant effect (with a large size), the paper itself reported no effect for either subgroups recruited (i.e. LBP patients with low or high disability) [54]. This inconsistency between the forest plot results and the original study could be due to the statistical model’s correction of variables for age effects. The same contrast was also obtained when LBP subgroups were compared separately with controls. In this case, people with LBP with low disability showed a significant effect. Overall, only one out of five studies reported a medium to large effect of the presence of LBP on movement variability during lifting tasks, characterized by a reduction in the predictability of lumbar movement.

#### Gait (walking and running)

**Fig 4:**
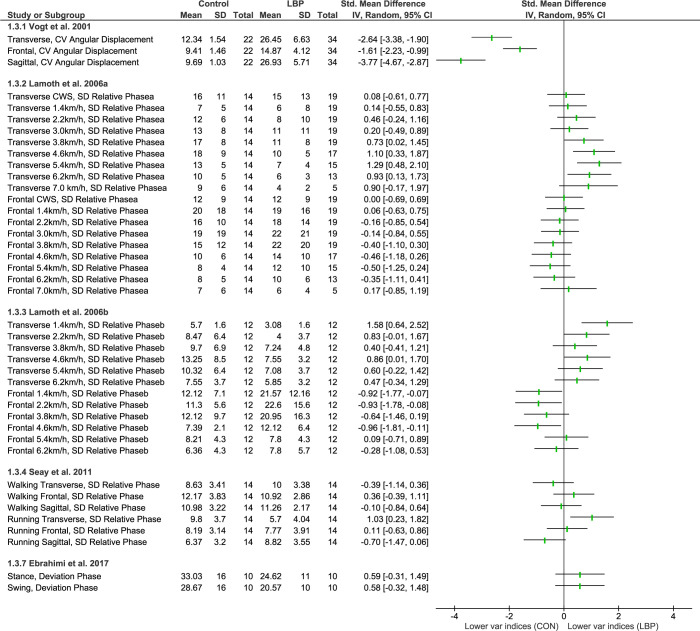
Differences in movement variability between people with LBP and healthy controls (CON) during walking or running tasks. Abbreviations: CV: Coefficient of Variation; SD: Standard deviation: Var: Variability.

Five studies included in this review used linear measures to quantify gait variability (**Fig 4).** One study [56] used angular displacement, while the other four studies [57–60] used a combination of angular displacement and velocity as coordination indices, specifically the continuous relative Fourier phase (RP), continuous relative phase (SD RP).

All five included studies reported some changes in at least one of their outcomes, with four studies finding a decrease [57–60] and two finding an increase in variability of gait (during walking or running) [56, 58]. Even though the forest plot showed no effects [60], the original article reported a significant decrease in variability. The discrepancy could be due to the authors’ use of a non-parametric test in their study since the data failed to fit a normal distribution. However, the most prominent finding was a decrease in variability of lumbar-pelvis coordination in the transverse plane and an increase in the frontal plane in those with LBP compared to controls regardless of walking speed [57, 58]. Overall, all studies reported a change in movement variability in people with LBP compared to healthy controls, with effect sizes ranging from medium to large.

#### Sit to stand to sit (STS) task

**Fig 5:**
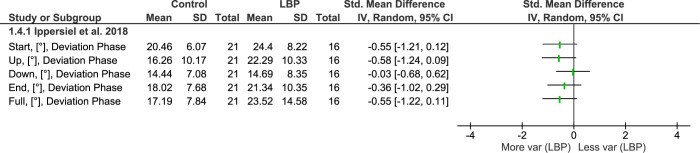
Differences in movement variability between people with LBP and healthy controls (CON) during sit to stand task. Abbreviations: SD: Standard deviation: Var: Variability.

Only one of the included studies was categorized under this task (**Fig 5)**. This study investigated movement variability during a repeated sit-to-stand-to-sit (STS) task [61], which divided the task into four periods: start, up, down, and end. The authors used DP to describe the amount of inter-joint coordination variability.

For the lumbar region, no effect of LBP status was observed over the full task. However, there was a significant period effect characterized by greater variability over the start and end period of the STS task. No group or interaction effect was observed.

### Methodological quality assessment

Five of the included studies [19, 50–52, 61] were rated as high-quality studies that met most of the quality criteria (75% to 83%) (**Table 4)**. On the other hand, nine studies [49, 53–60] partially met the quality criteria (67%) and were rated as a moderate quality studies. The two most common weaknesses occurred in the report of the treatment history of those with LBP (item 6), and sample size justification or power description (item 12), and less commonly, representativeness of LBP and control groups (item 8).

**Table 4:** Quality rating instrument using modified Downs and Black Scale (adjusted specifically for the current review).

*Studies*	*Item*												*Total/12*	*Percentage/100%*
	*Reporting*							*External validity*	*Internal validity/bias & confounding*			*Power*		
	1	2	3	4*	5*	6*	7	8	9	10	11*	12		
	*q*.*1 D&B*	*q*.*2 D&B*	*q*.*3D&B*				*q*.*7D&B*	*q*.*11 D&B*	*q*.*16 D&B*	*q*.*20 D&B*		*q*.*27 D&B*		
Graham et al. 2014 [49]	1	1	1	1	1	0	1	0	1	0	1	0	8	67
Mokhtarinia et al. 2016 [50]	1	1	1	1	1	***0***	1	0	1	1	1	0	9	75
Bauer et al. 2017 [51]	1	1	1	1	1	0	1	1	1	1	1	0	10	83
Dideriksen et al. 2014 [19]	1	1	1	1	1	0	1	1	1	0	1	0	9	75
Bauer et al. 2015 [52]	1	1	1	1	1	0	1	1	1	1	1	0	10	83
Moreno Catalá et al. 2018 [53]	1	1	1	1	1	0	1	0	1	0	1	0	8	67
Pranata et al., 2018 [54]	1	1	1	1	1	0	1	0	1	0	1	0	8	67
Fujii et al. 2022 [55]	1	1	1	1	1	0	1	0	1	***0***	0	1	8	67
Vogt et al. 2001 [56]	1	1	1	1	1	0	1	0	1	0	1	0	8	67
Lamoth et al. 2006a [57]	1	1	1	1	1	0	1	0	1	0	0	1	8	67
Lamoth et al. 2006b [58]	1	1	1	1	1	0	1	0	1	0	1	0	8	67
Seay JF et al. 2011 [59]	1	1	1	1	1	0	1	0	1	0	1	0	8	67
Ebrahimi et al. 2017 [60]	1	1	1	1	1	0	1	0	1	0	1	0	8	***67***
Ippersiel et al. 2018 [61]	1	1	1	1	1	0	1	1	1	1	1	***0***	10	83

### Level of evidence (GRADE)

The main outcome measures from individual studies were grouped per task type as domains and the obtained evidence was narratively described across studies. The quality of evidence per domain was summarised in accordance with GRADE and is reported in **Table 5**.

**Table 5:** Quality of evidence assessment based on Grading of Recommendations, Assessment, Development and Evaluation (GRADE) criteria.

Movement variability based on the task preformed	Number of LBP patients (studies)	Risk of bias (D&B scale score)	Inconsistency	Indirectness	Imprecision	Number of participants		Publication bias	Overall Quality of Evidence
						LBP group	Control group		
Repeated Flexion and Extension tasks	91 (3) Graham 2014 Mokhtarinia et al. 2016 Bauer et al. 2017	Not serious 67% 75% 83%	Not serious	Serious^b^ **↓**	Serious^c^ **↓**	10 22 59	10 22 27	Suspected^d^	Very low
Lifting tasks	184 (5) Dideriksen et al. 2014 Bauer et al. 2015 Moreno Catalá et al. 2018 Pranata et al. 2018 Fujii et al. 2022	Not serious 75% 83% 67% 67% 67%	Serious^a^ **↓**	Serious^b^ **↓**	Serious^c^ **↓**	17 63 30 43 31	17 31 29 29 20	Suspected^d^	Very low
Gait	89 (5) Vogt et al. 2001 Lamoth et al. 2006a Lamoth et al. 2006b Seay et al. 2011 Ebrahimi et al. 2017	Not serious 67% 67% 67% 67% 67%	Serious^a^ **↓**	Serious^b^ **↓**	Serious^c^ **↓**	34 19 12 14 10	22 14 12 14 10	Suspected^d^	Very low
Sit to stand to sit (STS) task	16 (1) Ippersiel et al. 2018	Not serious 83%	Serious^a^ **↓**	NA*	Serious^c^ **↓**	16	21	Suspected^d^	Very low

**GRADE:** High quality: very confident that the true effect lies close to that of the estimate of the effect and further research is very unlikely to change. Moderate quality: moderately confident in the effect estimate and further research may change the estimate. Low quality: limited confident in the effect estimate and further research is very likely to have an important impact to change the estimate. Very low quality: Any estimate of effect is very uncertain.

^**a**^ **↓**High level of heterogeneity; we downgrade one level due to variability in results or inconsistency between effects.

^**b**^ **↓**Evidence was not directly comparable to the question of interest; we downgrade one level due to the variety of outcome measurement tools including the used motion analysis systems and the analysis metrics.

^**c**^ **↓**Few participants and few events: we downgrade one level due to the wide confidence interval (CI) around the estimate of the effect (as estimated by forest plot).

^**d**^Limited number of observational studies with small sample size as well as asymmetrical distribution of observations on the funnel plot

NA* not applicable: Only one study.

The overall quality of evidence was downgraded from low to very low for all domains mostly due to issues concerning imprecision of the included studies, inconsistency between effects, and to a lesser extent due to indirectness and potential publication bias. Downgrading the quality of evidence based on imprecision, was addressed using the optimal information size rule (OIS) to present the required sample size for the continuous outcomes that was used in the included studies. The usual standards of α (0.05) and β (0.20), was used with an effect size of 0.2 standard deviations, representing a small effect, which required a total sample size of approximately 400 (200 per group) [64]. However, the included studies had a maximum sample size of 94 participants, which suggests a small sample size, and resulted in a wide confidence interval (CI) around the estimate of the effect as detected by the forest plot. With regards to inconsistency, all domains showed serious concerns in heterogeneity of the results except for the repeated flexion and extension tasks that presented a significant overlap of confidence intervals as observed in the forest plot, suggesting minimal variation in effect sizes.

## Discussion

This review is the first to provide a comprehensive analysis of observational studies that investigated whether thoraco-lumbar and lumbo-pelvic kinematic variability is altered in people with non-specific chronic LBP compared to asymptomatic individuals, while preforming repetitive cyclic functional tasks. Asymptomatic people typically exhibit a normal range of motor variability when performing functional tasks [65], however, those with LBP may display motor adaptations resulting in altered movement variability [66]. Movement variability is known to range from complete repeatability to excessive variability in both space and time [23]. However, the degree of adaptive variability in the trunk during repetitive functional tasks among individuals with LBP is not well established which emphasises the need to synthesise the literature for a better understanding of the optimal adaptive variability for people with chronic LBP.

To facilitate interpretation, this review grouped the results into four categories based on the tasks used to investigate movement variability; repetitive trunk flexion and extension, lifting, gait, and in one study, sit-to-stand-to-sit. While the overall quality of evidence in this review was rated as very low, due to the inclusion criteria of observational studies with small sample sizes, all included studies were of high to medium methodological quality, with no study rated as low quality. However, heterogeneity in the metrics used for analysis and effect sizes contributed to downgrade the quality of evidence.

Although this review sub-grouped the evidence for better interpretation, the choice of metrics within each group was crucial for understanding changes in movement variability. As mentioned previously, linear and non-linear metrics capture distinct aspects of movement variability that may vary independently from one another [23]. For example, an increase in the structure of variability does not necessarily indicate an increase in the amount of variability. In the reviewed studies, changes in the structure of variability were commonly described using terms such as predictability or repeatability, rather than an increase or decrease in variability. Conversely, the amount of variability was typically reported simply an increase or decrease.

### Differences in movement variability between people with and without chronic LBP

This review found that people with chronic non-specific LBP move their lumbar spine in a complex and less predictable way, likely as an adaptive response to pain, particularly noted as a change in the angular velocity of the trunk during repetitive flexion and extension [49–51]. Similarly, the task of lifting showed a general reduction in the predictability of angular displacement, velocity and acceleration [19, 52–55]. Although this result was drawn from non-linear metrics, for a comparable result, less predictable behaviour can be indirectly described as an increase in the amount of variability. The nature of both tasks similarly involves predetermined (loaded/unloaded, symmetrically/ asymmetrically) repetitive trunk movements which resulted in people with LBP moving their lumbar spine in a more variable. This finding is consistent with a previous review that found a common feature among individuals with chronic or mechanical LBP was increased kinematic spinal movement variability [21]. Despite this consistency, these findings should be interpreted with caution due to the heterogeneity of the metrics used, which are conceptually different [20]. Moreover, the level of evidence supporting this observation is very low, indicating a lack of confidence in the estimate of this result.

Given that walking is one of the most common activities of daily living, numerous studies have examined motor variability during gait [27]. Our review found that people with chronic non-specific LBP experience changes in kinematic variability during gait regardless of walking speed, which was primarily in coordination patterns of movement; however, the direction of change was not consistent [56–60]. Gait studies typically investigate motor adaptations across multiple planes of movement, which provide a more comprehensive understanding of the direction of motor control changes. For instance, the reviewed studies reported more rigid and less variable coordination between transverse lumbo-pelvic rotations in people with LBP compared to healthy controls, whereas the coordination between spinal regions in the frontal plane was less rigid and more variable. This finding aligns with a previous review that found altered motor control in people with chronic LBP during gait, showing higher stride-to-stride kinematic variability. However, this was not surprising considering that most of the studies included in the previous review were also eligible for our review [26]. The level of evidence was rated very low, although the evidence was based on linear metrics of variability only, this was mostly due to the small sample sizes that led to wide CIs around the estimate of the effect.

There was limited evidence examining the sit-to-stand-to-sit task. However, similar to other tasks such as gait, the evidence was based on the amount of inter-joint coordination variability. Yet, no difference was observed in the adaptive variability during task performance between people with and without chronic LBP [61].

### LBP characteristics and movement variability

This review analysed 14 observational studies with a total of 380 participants with non-specific chronic LBP to determine the level of adaptive variability during various repetitive functional activities. Although the type of LBP was predetermined as chronic non-specific, there was no restrictions on LBP duration, intensity, or level of disability. However, the level of both LBP intensity and disability was minimal across studies, and some of the functional tasks may not have been challenging enough to evoke noticeable changes in movement variability. Although previous research has established a connection between pain-related threat beliefs and disability with trunk kinematics in people with LBP, there is a scarcity of research exploring the impact of factors like fear-avoidance behaviour/beliefs and catastrophic thinking on kinematic variability [67]. Only one study in the current review investigated the correlation between pain-related fear and trunk inter-joint coordination variability in people with chronic LBP [55]. Therefore, further studies are needed to explore the relationship between movement variability and pain-related psychological factors.

### Methodological considerations

The current review adhered to rigorous methodology outlined in a predefined and published protocol [32]. Screening, quality assessment, and data extraction was performed independently by two reviewers. In addition, unlike previous scoping reviews that did not assess the overall level of evidence [20, 21], the current review utilized GRADE to evaluate the overall level of evidence. Although meta-analysis was not applicable, we presented our findings quantitatively using forest plots to support the narrative synthesis.

There are some limitations that should be acknowledged. The current review included a limited number of observational studies with small sample sizes, which could have led to potential publication bias. Additionally, there are no established recommendations for evidence synthesis of observational studies, which makes it challenging to draw definitive conclusions based on the observational nature of the presented evidence [68].

## Conclusion

Despite very low-level evidence, people with chronic non-specific LBP exhibited altered motor adaptability, as indicated by differences in kinematic movement variability during repetitive functional tasks. However, the direction of these changes in movement variability was not consistent across individuals with chronic LBP. This systematic review highlights the importance of standardizing terminology for assessing kinematic variability and emphasizes the need for future research to consider the conceptual differences between different metrics.

## Supporting information

S1 Table Preferred reporting items for systematic reviews and meta-analyses checklist-PRISMA. (DOCX)

S2 Table Inclusion and the exclusion criteria of the included studies. (PDF)

S3 Table Detailed description of the task preformed in the included studies. (PDF)

S1 File Search strategy used in MEDLINE (OVID interface). (PDF)

S2 File Excluded studies with reasons. (XLSX)
