# Supplementary material for: Is movement variability altered in people with chronic non-specific low back pain? A systematic review
Source: PLoS One. 2023 Jun 14;18(6):e0287029. doi: 10.1371/journal.pone.0287029 (PMC10266636; doi:10.1371/journal.pone.0287029)
Supplement: S2 Table — (PDF) [file pone.0287029.s002.pdf]

**S4 Table.** Inclusion/exclusion criteria of included studies	
**First Author**	**Inclusion/exclusion criteria**
**1. Repeated Flexion and Extension task**	
Graham et al. 2014[48]	*Inclusion criteria:*<br>• Continuous non-specific LBP ≥ four weeks<br>• Treated medically and/or missed game time due to their back pain<br>• Healthy participants were included if they experienced LBP that lasted for longer than four weeks, had never missed game time due to LBP, and had never received treatment for LBP
Mokhtarinia et al. 2016[49]	*Inclusion criteria:*<br>• Episodic LBP ≥12 months with limited function due to the back pain<br>*Exclusion criteria:*<br>• Structural deformities of the spine<br>• Pain extending to and/or beyond the gluteal fold<br>• Previous spine, hip or knee surgery<br>• Spinal root pain.<br>• Spinal tumors or infections<br>• Neurologic symptoms such as weakness, tingling or anesthesia in lower extremities<br>• Visual or hearing impairment
Bauer et al. 2017 [50]	*Inclusion criteria:*<br>• Age (18 to 65) years<br>• A current episode of sub-acute or chronic non-specific LBP ≥ four weeks<br>• A low level of psychosocial risk factors<br>*Exclusion criteria*<br>• Specific LBP<br>• Vertigo or disturbance of the equilibrium.<br>• Systemic diseases.<br>• Pain in other areas of the body.<br>• Complaints, injury, or surgery of the legs (hips to feet) within the last six months<br>• Medication affecting postural control (e.g. anti-depressants)<br>• Pregnancy
**2. Lifting task**	

Dideriksen et al. 2014[19]	*Inclusion criteria:*<br>• Aged between 18 and 45 years<br>• Subjects with non-specific episodic LBP (> three months) Each episode of LBP (within last six months) should have lasted > one week with sufficient intensity to limit function<br>*Exclusion criteria:*<br>• Any major circulatory, neurological, or respiratory disorders<br>• Recent or current pregnancies<br>• Previous spinal surgery<br>• Current treatment for LBP from health care providers, or participation in specific trunk muscle exercise in the past 3 months<br>• Taking any medication such as opioids, anticonvulsants, or antidepressants<br>• Taking non-steroidal anti-inflammatory drugs (NSAIDs) on a regular basis
Bauer et al. 2015[51]	Same as Bauer et al. 2017 [50]
Moreno Catalá et al. 2018[52]	*Inclusion criteria:*<br>• Chronic non-specific back pain within the last 12 weeks<br>• Evidence of LBP induced limitations during daily activities.<br>*Exclusion criteria:*<br>• history of spinal operation, prolapse, herniated disks, arthritis, mental, neurological, or cardiovascular diseases, sensorimotor deficits, abnormal spinal column structural changes.<br>• Continuous dependency of pain relieve medication<br>• Restrictions from participating in sporting activity from a doctor<br>• Undergoing physiotherapist treatment
Pranata et al., 2018[53]	*Inclusion criteria:*<br>• Reported pain between the level of the twelfth thoracic vertebra (T12) and the gluteal fold<br>• Pain persisted for >3 months<br>*Exclusion criteria:*<br>• If they presented with overt neurological signs; muscle weakness, previous spinal surgery, systemic or inflammatory conditions such as rheumatoid arthritis, malignancy, unstable spondylolisthesis (i.e., specific diagnosis of chronic LBP)<br>• Inability to understand written or spoken English
Fujii et al., 2022[54]	*Inclusion criteria:*<br>• Male workers aged 20–40 years.<br>• LBP duration of > 3 months<br>• Score ≥ 1 on a NRS for pain intensity during work in the past 4 weeks<br>*Exclusion criteria:*<br>• Previous diagnosis of spinal disease (lumbar disc herniation, lumbar spondylolisthesis, or lumbar osteoarthritis).<br>• Pain in peripheral joints in an upper or lower limb<br>• Presence of neurological symptoms of a lower limb

	• Serious spinal pathology (cancer, inflammatory arthropathy, or acute vertebral fracture), or a diagnosis of neurological disease
**3. Gait**	
Vogt et al. 2001[55]	*Inclusion criteria:*<br>• Idiopathic chronic LBP without nerve root tension, neurologic deficits, or previous surgery of the spine or lower extremities<br>• experienced low back pain on at least half the days in the past 12 months in a single or multiple episodes<br>• The pain between T12 and the gluteal folds<br>*Exclusion criteria:*<br>• Patients with joint abnormalities of the lower limbs, pregnancy, vascular insufficiency, or systemic problems (e.g., cancerous, cardiovascular, or endocrinologic diseases)<br>• Left and right leg lengths were not allowed to show more than 1 cm of inequality
Lamoth et al. 2006a[56]	*Inclusion criteria:*<br>• Medical diagnosis of non-specific LBP with pain and symptoms persisting > 3 months for which medical treatment had been sought<br>• Age between 18 and 65 years<br>• Ambulation without a walking aid<br>*Exclusion criteria:*<br>• LBP of traumatic or structural origin<br>• LBP with neurological symptoms or pain radiation in the lower leg(s)<br>• previous back surgery,<br>• Spinal tumors or infections<br>• neurological and/or musculoskeletal disorders unrelated to LBP
Lamoth et al. 2006b[57]	Same as Lamoth et al., 2006a [56]
Seay JF et al. 2011[58]	*Inclusion criteria:*<br>• Recreational runners with at least 20 km/week running experience<br>• Ages 18–40<br>• The LBP group experienced LBP for > 4 months<br>• The remaining group had never experienced LBP<br>*Exclusion criteria:*<br>• LBP as a result of traumatic injury<br>• Medically diagnosed with a slipped or bulging inter-vertebral disc, and/or if they reported symptoms of neurological involvement such as numbness and tingling in the extremities or down the backside of the pelvis and thigh
Ebrahimi et al. 2017[59]	*Inclusion criteria:*<br>• Subjects age (18-40 yrs)<br>• > 12 weeks LBP with unknown origin<br>• NRS between 4 and 6 points averaged in the last 7 days<br>• Their disability level based (ODI) ranged from 21% to 60%.

	Exclusion criteria:<br>• Any spinal or lower limbs' deformity, infection or tumors, any orthopedic/neurologic or sensory disorders, rheumatoid disease, history of lower limbs, trunk or pelvic fractures or surgical interventions<br>• Radicular pain to lower limbs, spondylolisthesis, balance disorders.<br>• Received physical therapy in the past three months and had taken anti-inflammatory/analgesic medicine in the past 48 h
**4. Sit to stand to sit (STS) task**	
Ippersiel et al. 2018[60]	*Inclusion criteria:*<br>• Had pain primarily located between the gluteal folds and ribs<br>*Exclusion criteria:*<br>• Evidence of spinal stenosis or radiculopathy, any serious underlying condition (cauda equina syndrome, cancer, infection), or another specific spinal condition (vertebral compression fracture, ankylosing spondylitis)<br>• Neurological or respiratory conditions that might affect STS, major postural abnormality (e.g. scoliosis of $\geq 7°$)<br>• Previous spinal surgery or trauma (e.g. fracture)<br>• Pregnancy in the past 2 years
