# Supplementary material for: Is movement variability altered in people with chronic non-specific low back pain? A systematic review
Source: PLoS One. 2023 Jun 14;18(6):e0287029. doi: 10.1371/journal.pone.0287029 (PMC10266636; doi:10.1371/journal.pone.0287029)
Supplement: S3 Table — (PDF) [file pone.0287029.s003.pdf]

**S5 Table**. Task description of included studies	
**First Author**	**Task preformed**
**1. Repeated Flexion and Extension task**	
Graham et al. 2014[49]	Two randomized trials, symmetric and asymmetric, of 30 repetitive and continuous trunk flexion and extension movements from standing position, with a constrained pelvis and their hands held together while touching instrumented targets at specified locations The movement pace was 15 cycles per minute, or 4 s per cycle.
Mokhtarinia et al. 2016[50]	30 cycles of repeated trunk flexion–extension (touching target placed at knee level followed by returning to upright position) in time with a metronome under various conditions of trunk *asymmetry* (symmetric: target placed at sagittal midline; asymmetric: target placed 60º to the right of mid-sagittal plane inducing right trunk rotation), *velocity* (low: 20 cycles per minute; high: 40 cycles per minute), and *loading* (with or without wearing 8 kg loaded vest)
Bauer et al. 2017[51]	Repeated trunk flexion and extension test in sitting position, 20 cycles, 3 sec. per cycle, 60 sec. total in duration, 1 repetition. Speed controlled by metronome set at 80 bpm
**2. Lifting task**	
Dideriksen et al. 2014[19]	Repetitively move a box (5 kg) between two shelves, located at knee and shoulder height; 22 cycles, 8 second per cycle, 3 min
Bauer et al. 2015[52]	Pick up a box from standing position, 10 cycles, 4 sec duration, 1 repetition, (box 10% of body weight located at a standardized stance in front of the participant)
Moreno Catalá et al. 2018[53]	Lifting task, in which a pot (1.5 kg) was cyclically moved back and forth between two tables of different heights (40 cycles); participant standing in the middle of two tables (positioned 90° to each other) to induce 45° trunk rotation; velocity: 12 cycles per minute induced by a metronome; total of 40 cycles
Pranata et al., 2018[54]	Lifting an 8-kg kettlebell up to the level of their abdomen using a self-selected pace and technique; repeated twice but only one trial analysed
Fujii et al., 2022[55]	Lifting a box (520 × 365 × 305 mm) placed on the ground as quickly as possible to waist-height. In three different conditions: The weight of the box 10%, 30%, or 50% of the subject's body weight (preformed five times for each weight condition)
**3. Gait**	

Vogt et al. 2001[56]	Walking on a motorized treadmill at 4.5 km/h; data from a 30-second interval recorded while walking for approximately 3 minutes
Lamoth et al. 2006a[57]	Walking on a treadmill at a self-selected walking velocity succeeded by increasing velocity from 1.4 km/h to a maximally attainable walking velocity of up to 7.0 km/h (with increments of 0.8 km/h)
Lamoth et al. 2006b[58]	Walking on a treadmill at six velocities in a fixed order: 6.2, 1.4, 3.8, 5.4, 2.2, and 4.6 km/h; Recordings started immediately lasted 30s for each velocity
Seay JF et al. 2011[59]	Walking (at various speeds systematically increased from 2.88km/h in increments of 1.8km/h to 13.68 km/h, each stage lasted 30s and data collected for the last 20s followed by running (at preferred speed)
Ebrahimi et al. 2017[60]	Walking barefoot along an 8-m walkway at a comfortable self-selected speed; at least 20 complete gait cycles for each limb captured
**4. Sit to stand to sit (STS) task**	
Ippersiel et al. 2018[61]	Sit to stand to sit (STS) task with arms crossed over chest, including standing upright from sitting and return to sitting as quickly as possible; divided into 4 sequential periods including ‘start’ (from the point of greatest backward motion of the centre of motion to the point of loss of contact with the seat), ‘up’ (end of start to the point of upright standing), ‘down’ (from upright standing to the point of contact with the seat) and ‘end’ (from point of contact with the seat to the point of greatest backward motion of the centre of pressure); 10 trials
