# Supplementary material for: Is movement variability altered in people with chronic non-specific low back pain? A systematic review
Source: PLoS One. 2023 Jun 14;18(6):e0287029. doi: 10.1371/journal.pone.0287029 (PMC10266636; doi:10.1371/journal.pone.0287029)
Supplement: S1 File — (PDF) [file pone.0287029.s004.pdf]

<u>Search Strategy:</u>

MEDLINE

1. ((lumba* or low* back) adj3 (pain* or ach* or injur*)).mp.
2. exp Low Back Pain/
3. (lumbago or lumbalgia).mp.

4. 1 or 2 or 3

5. (trunk or torso or back or spine).mp.
6. exp Lumbosacral Region/
7. Thoracolumbar.mp.
8. exp Spine/

9. 5 or 6 or 7 or 8

10. ((movement or motion) adj2 (Varia* or Quality or Complexit* or Irregularit* or Consistenc* or Inconsistenc* or Stabil* or kinematic* or Coordination)).mp.
11. Mechanical coupling.mp.
12. exp motor control/

13. 10 or 11 or 12

14. 4 and 9 and 13

15. limit 14 to (english language and humans)
